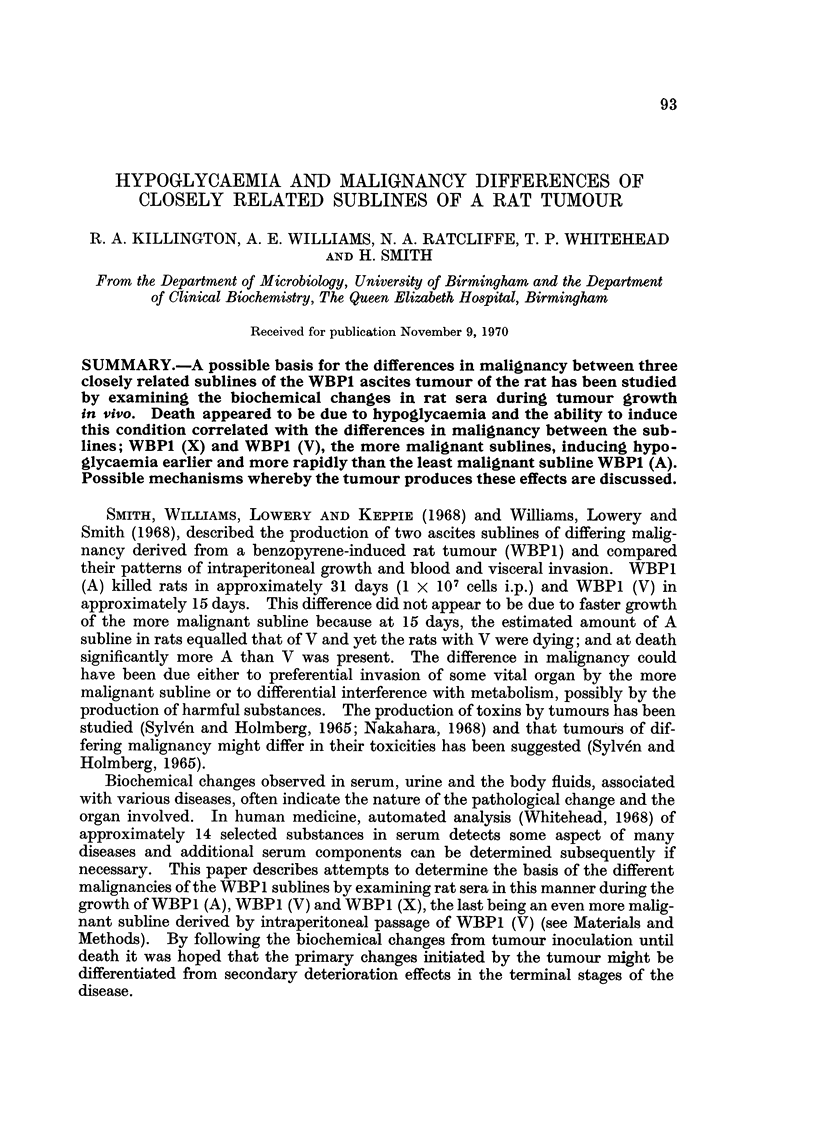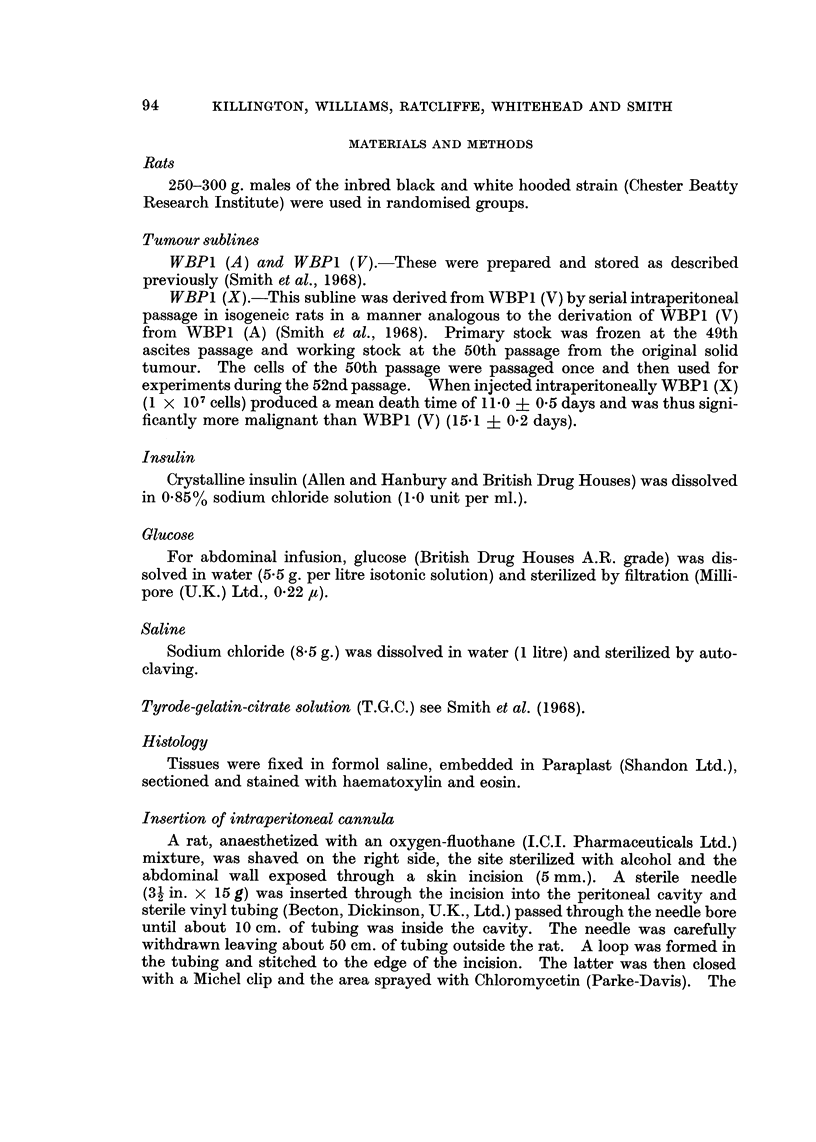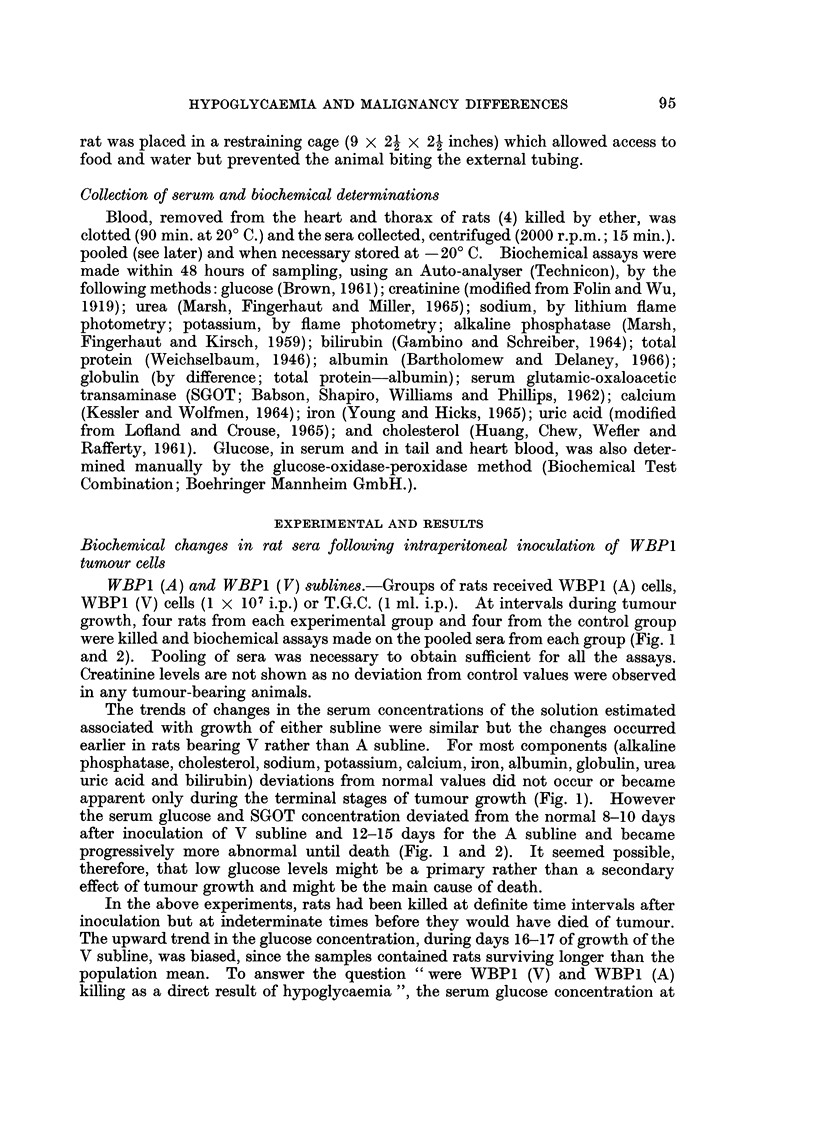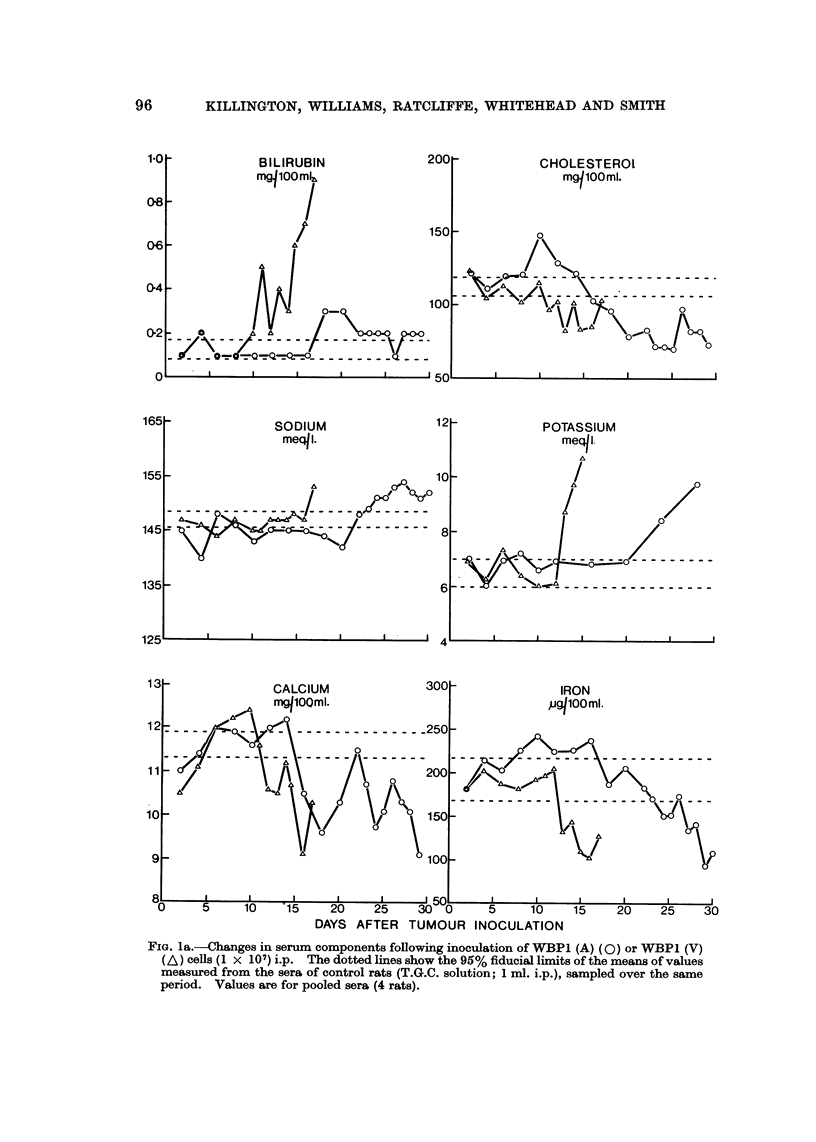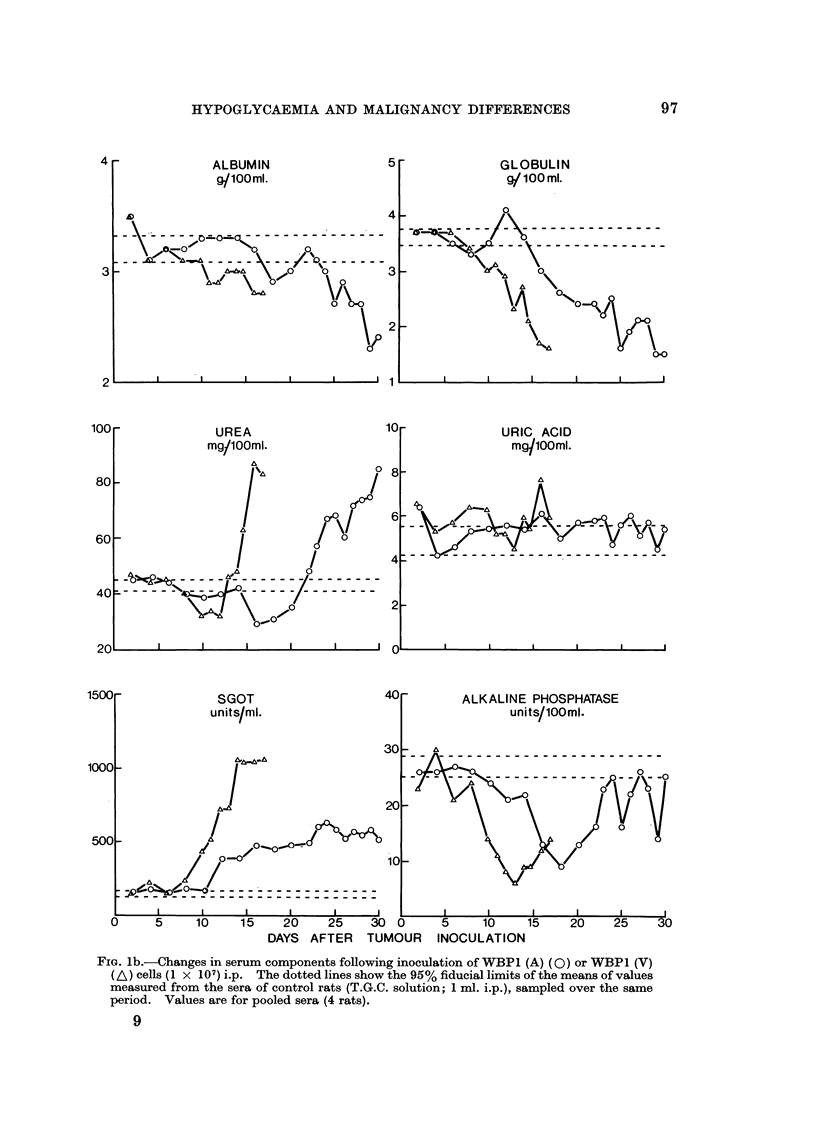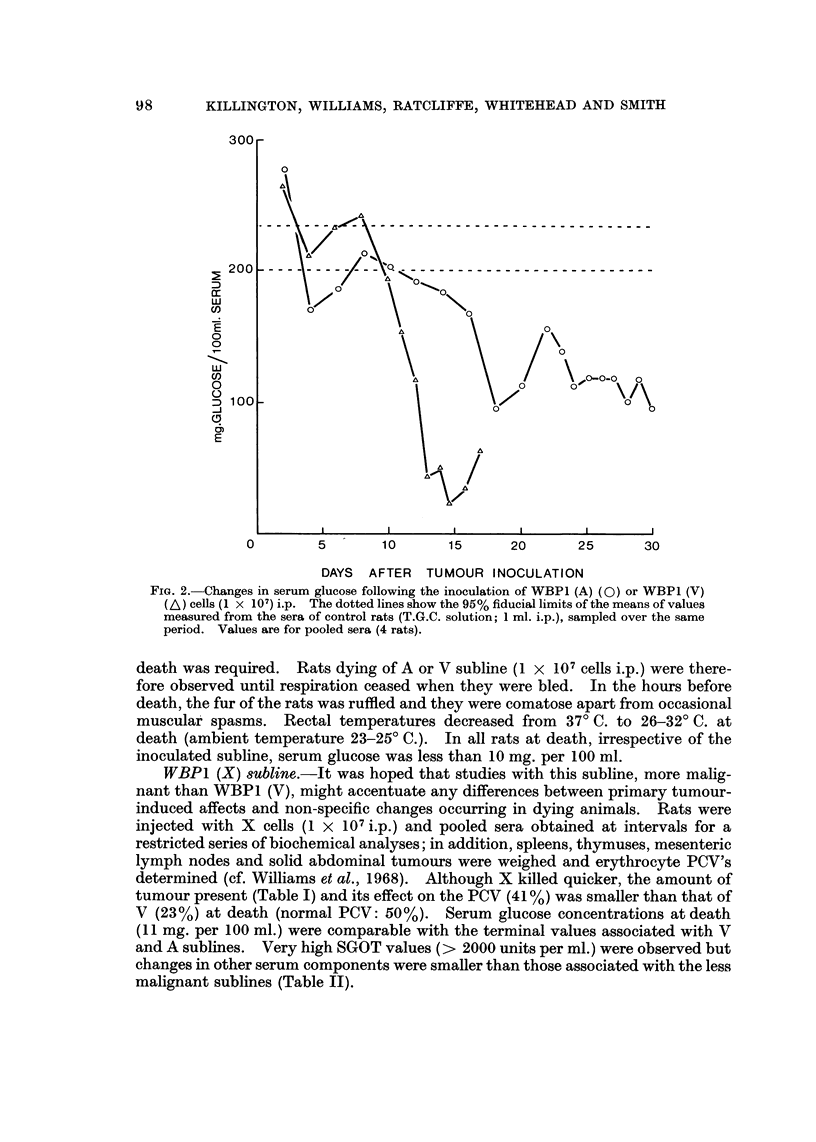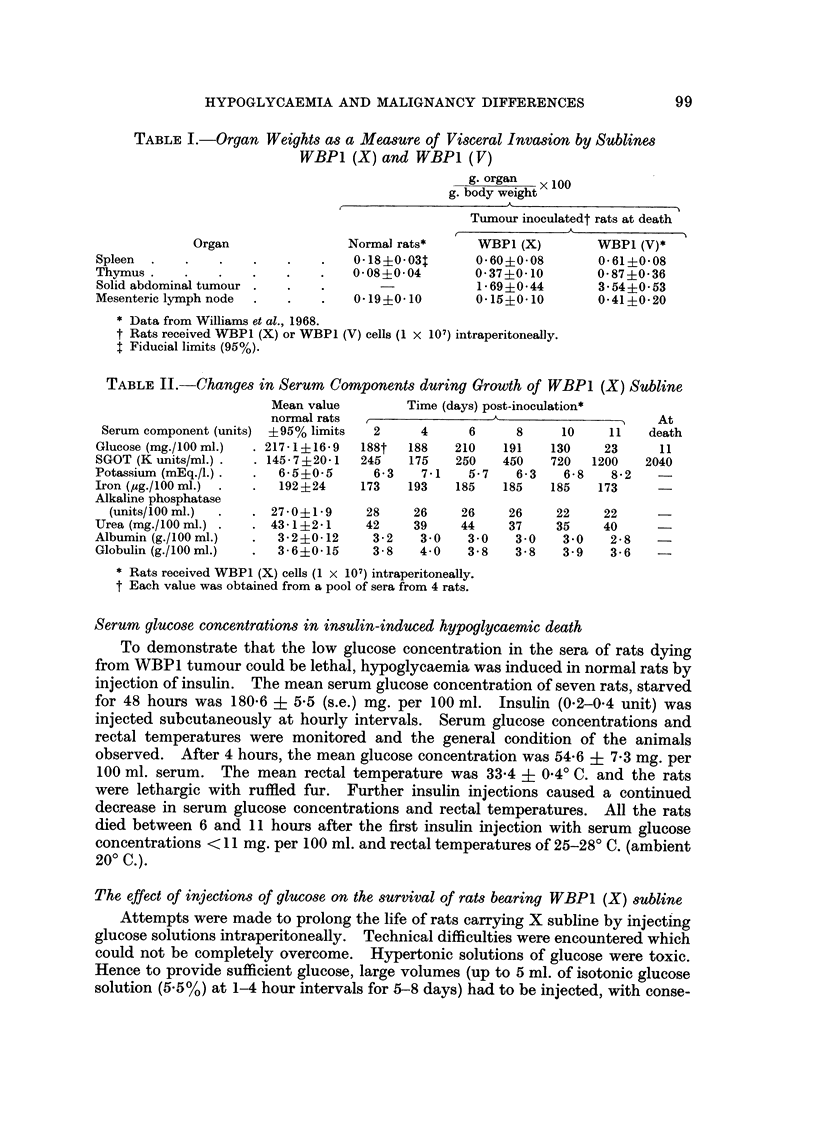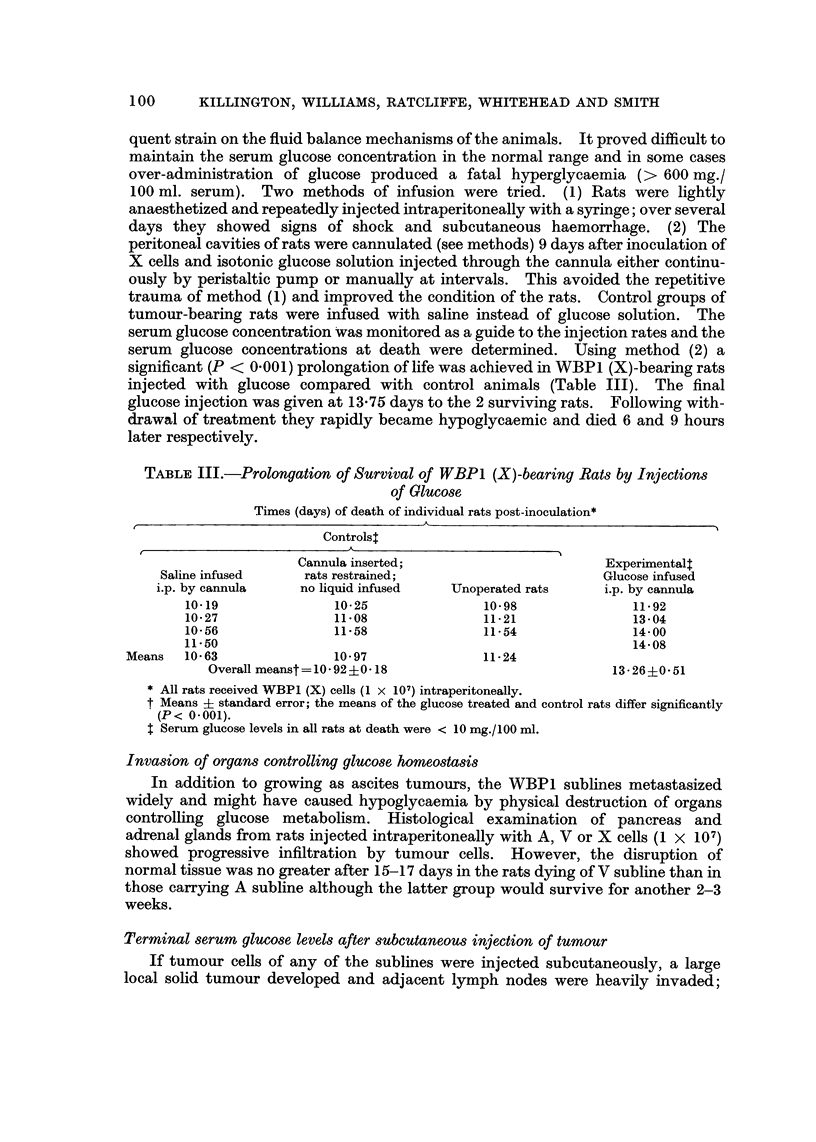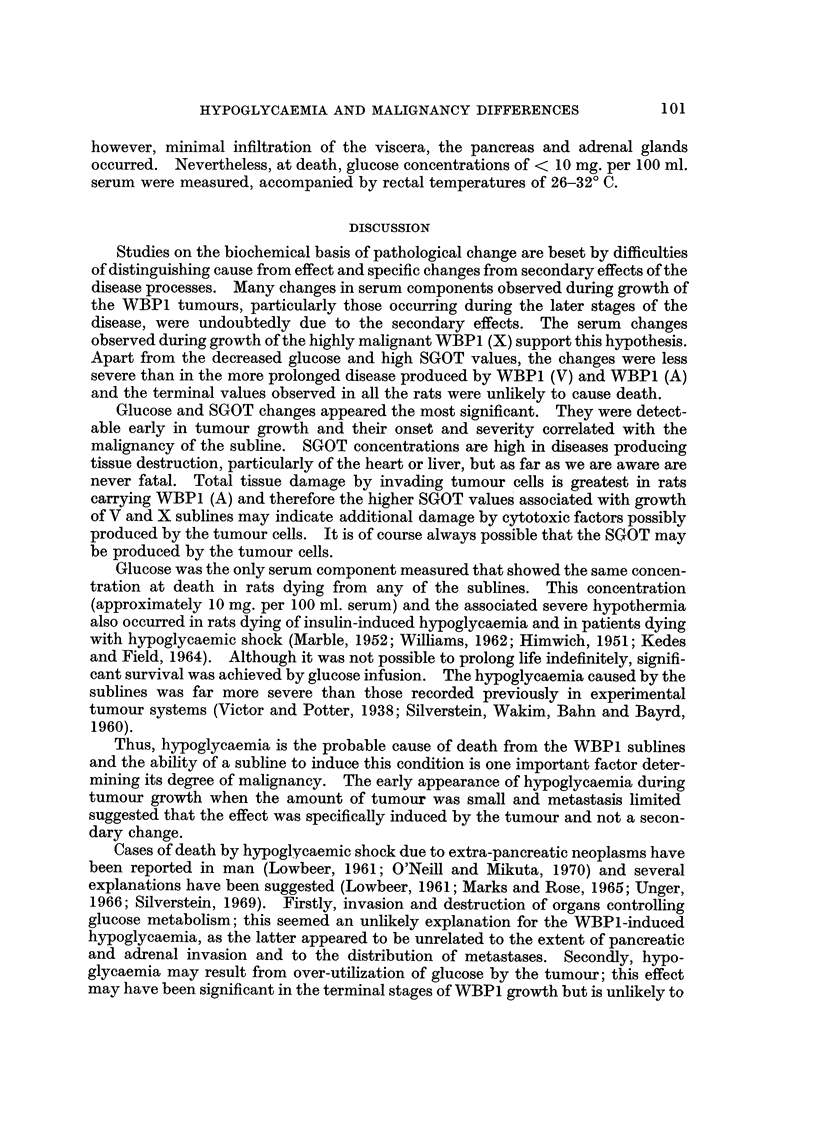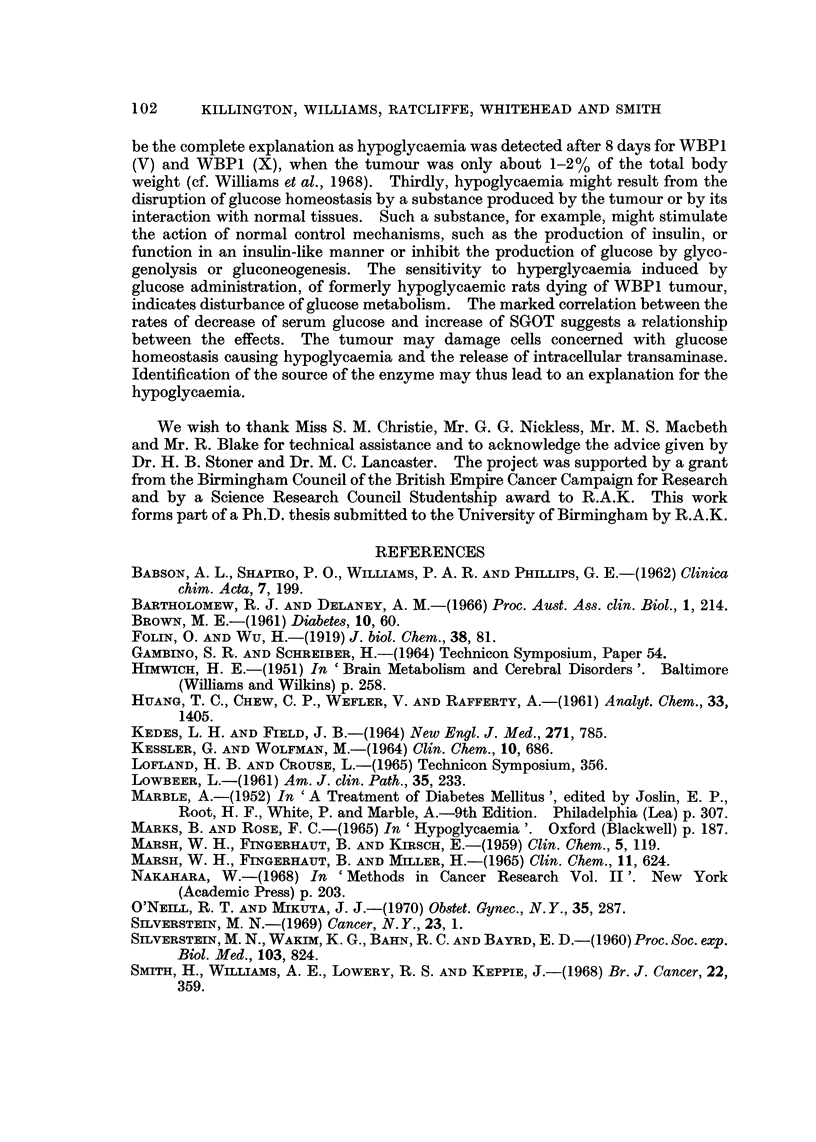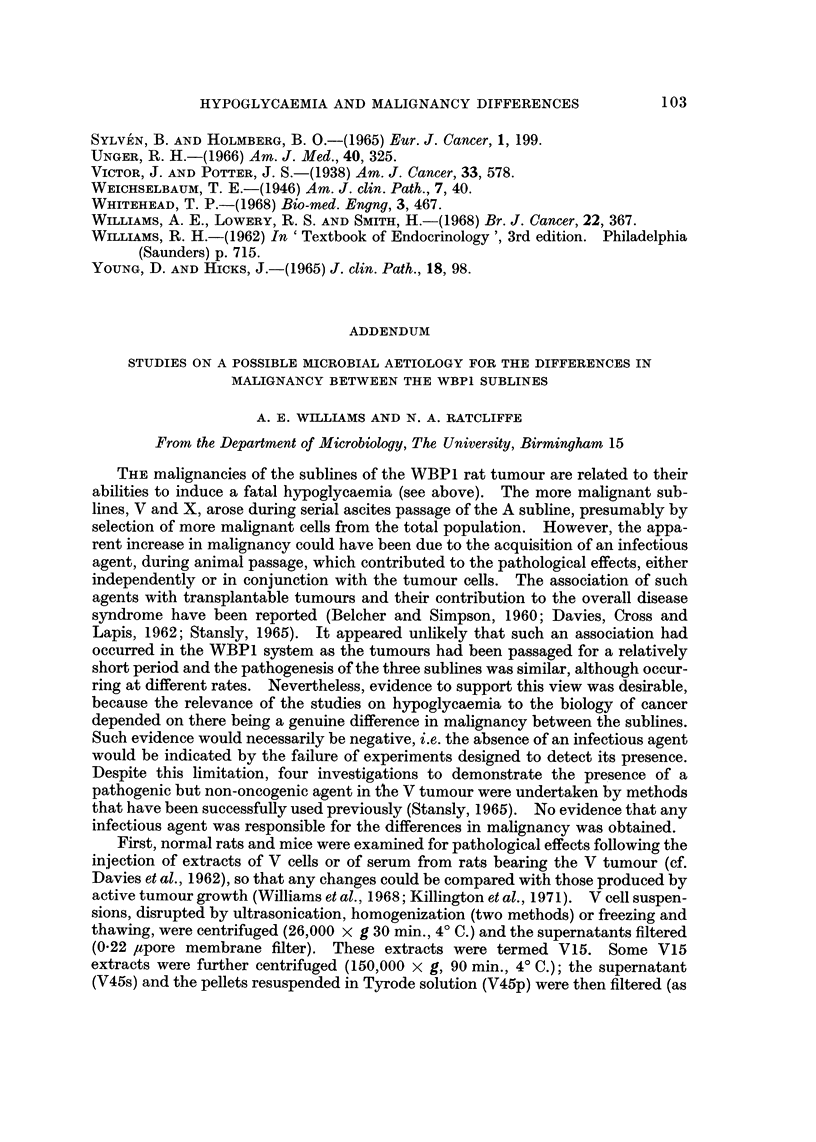# Hypoglycaemia and Malignancy Differences of Closely Related Sublines of a Rat Tumour

**DOI:** 10.1038/bjc.1971.13

**Published:** 1971-03

**Authors:** R. A. Killington, A. E. Williams, N. A. Ratcliffe, T. P. Whitehead, H. Smith

## Abstract

A possible basis for the differences in malignancy between three closely related sublines of the WBP1 ascites tumour of the rat has been studied by examining the biochemical changes in rat sera during tumour growth *in vivo.* Death appeared to be due to hypoglycaemia and the ability to induce this condition correlated with the differences in malignancy between the sublines; WBP1 (X) and WBP1 (V), the more malignant sublines, inducing hypoglycaemia earlier and more rapidly than the least malignant subline WBP1 (A). Possible mechanisms whereby the tumour produces these effects are discussed.


### HYPOGLYCAEMIA AND MALIGNANCY DIFFERENCES OF CLOSELY RELATED SUBLINES OF A RAT TUMOUR

R. A. KILLINGTON, A. E. WILLIAMS, N. A. RATCLIFFE, T. P. WHITEHEAD AND H. SMITH

*From the Department of Microbiology, University of Birmingham and the Department of Clinical Biochemistry, The Queen Elizabeth Hospital, Birmingham*

Received for publication November 9, 1970

**SUMMARY.—A possible basis for the differences in malignancy between three closely related sublines of the WBP1 ascites tumour of the rat has been studied by examining the biochemical changes in rat sera during tumour growth *in vivo*. Death appeared to be due to hypoglycaemia and the ability to induce this condition correlated with the differences in malignancy between the sublines; WBP1 (X) and WBP1 (V), the more malignant sublines, inducing hypoglycaemia earlier and more rapidly than the least malignant subline WBP1 (A). Possible mechanisms whereby the tumour produces these effects are discussed.**

SMITH, WILLIAMS, LOWERY AND KEPPIE (1968) and Williams, Lowery and Smith (1968), described the production of two ascites sublines of differing malignancy derived from a benzopyrene-induced rat tumour (WBP1) and compared their patterns of intraperitoneal growth and blood and visceral invasion. WBP1 (A) killed rats in approximately 31 days ($1 \times 10^7$ cells i.p.) and WBP1 (V) in approximately 15 days. This difference did not appear to be due to faster growth of the more malignant subline because at 15 days, the estimated amount of A subline in rats equalled that of V and yet the rats with V were dying; and at death significantly more A than V was present. The difference in malignancy could have been due either to preferential invasion of some vital organ by the more malignant subline or to differential interference with metabolism, possibly by the production of harmful substances. The production of toxins by tumours has been studied (Sylvén and Holmberg, 1965; Nakahara, 1968) and that tumours of differing malignancy might differ in their toxicities has been suggested (Sylvén and Holmberg, 1965).

Biochemical changes observed in serum, urine and the body fluids, associated with various diseases, often indicate the nature of the pathological change and the organ involved. In human medicine, automated analysis (Whitehead, 1968) of approximately 14 selected substances in serum detects some aspect of many diseases and additional serum components can be determined subsequently if necessary. This paper describes attempts to determine the basis of the different malignancies of the WBP1 sublines by examining rat sera in this manner during the growth of WBP1 (A), WBP1 (V) and WBP1 (X), the last being an even more malignant subline derived by intraperitoneal passage of WBP1 (V) (see Materials and Methods). By following the biochemical changes from tumour inoculation until death it was hoped that the primary changes initiated by the tumour might be differentiated from secondary deterioration effects in the terminal stages of the disease.

### MATERIALS AND METHODS

### *Rats*

250–300 g. males of the inbred black and white hooded strain (Chester Beatty Research Institute) were used in randomised groups.

### *Tumour sublines*

*WBP*1 (*A*) *and WBP*1 (*V*).—These were prepared and stored as described previously (Smith *et al.*, 1968).

*WBP*1 (*X*).—This subline was derived from WBP1 (V) by serial intraperitoneal passage in isogeneic rats in a manner analogous to the derivation of WBP1 (V) from WBP1 (A) (Smith *et al.*, 1968). Primary stock was frozen at the 49th ascites passage and working stock at the 50th passage from the original solid tumour. The cells of the 50th passage were passaged once and then used for experiments during the 52nd passage. When injected intraperitoneally WBP1 (X) ($1 \times 10^7$ cells) produced a mean death time of $11{\cdot}0 \pm 0{\cdot}5$ days and was thus significantly more malignant than WBP1 (V) ($15{\cdot}1 \pm 0{\cdot}2$ days).

### *Insulin*

Crystalline insulin (Allen and Hanbury and British Drug Houses) was dissolved in 0·85% sodium chloride solution (1·0 unit per ml.).

### *Glucose*

For abdominal infusion, glucose (British Drug Houses A.R. grade) was dissolved in water (5·5 g. per litre isotonic solution) and sterilized by filtration (Millipore (U.K.) Ltd., 0·22 $\mu$).

### *Saline*

Sodium chloride (8·5 g.) was dissolved in water (1 litre) and sterilized by autoclaving.

*Tyrode-gelatin-citrate solution* (T.G.C.) see Smith *et al.* (1968).

### *Histology*

Tissues were fixed in formol saline, embedded in Paraplast (Shandon Ltd.), sectioned and stained with haematoxylin and eosin.

### *Insertion of intraperitoneal cannula*

A rat, anaesthetized with an oxygen-fluothane (I.C.I. Pharmaceuticals Ltd.) mixture, was shaved on the right side, the site sterilized with alcohol and the abdominal wall exposed through a skin incision (5 mm.). A sterile needle ($3\frac{1}{2}$ in. $\times$ 15 *g*) was inserted through the incision into the peritoneal cavity and sterile vinyl tubing (Becton, Dickinson, U.K., Ltd.) passed through the needle bore until about 10 cm. of tubing was inside the cavity. The needle was carefully withdrawn leaving about 50 cm. of tubing outside the rat. A loop was formed in the tubing and stitched to the edge of the incision. The latter was then closed with a Michel clip and the area sprayed with Chloromycetin (Parke-Davis). The rat was placed in a restraining cage (9 × 2½ × 2½ inches) which allowed access to food and water but prevented the animal biting the external tubing.

*Collection of serum and biochemical determinations*

Blood, removed from the heart and thorax of rats (4) killed by ether, was clotted (90 min. at 20° C.) and the sera collected, centrifuged (2000 r.p.m.; 15 min.). pooled (see later) and when necessary stored at −20° C. Biochemical assays were made within 48 hours of sampling, using an Auto-analyser (Technicon), by the following methods: glucose (Brown, 1961); creatinine (modified from Folin and Wu, 1919); urea (Marsh, Fingerhaut and Miller, 1965); sodium, by lithium flame photometry; potassium, by flame photometry; alkaline phosphatase (Marsh, Fingerhaut and Kirsch, 1959); bilirubin (Gambino and Schreiber, 1964); total protein (Weichselbaum, 1946); albumin (Bartholomew and Delaney, 1966); globulin (by difference; total protein—albumin); serum glutamic-oxaloacetic transaminase (SGOT; Babson, Shapiro, Williams and Phillips, 1962); calcium (Kessler and Wolfmen, 1964); iron (Young and Hicks, 1965); uric acid (modified from Lofland and Crouse, 1965); and cholesterol (Huang, Chew, Wefler and Rafferty, 1961). Glucose, in serum and in tail and heart blood, was also determined manually by the glucose-oxidase-peroxidase method (Biochemical Test Combination; Boehringer Mannheim GmbH.).

### EXPERIMENTAL AND RESULTS

*Biochemical changes in rat sera following intraperitoneal inoculation of WBP1 tumour cells*

*WBP1 (A) and WBP1 (V) sublines.*—Groups of rats received WBP1 (A) cells, WBP1 (V) cells (1 × $10^7$ i.p.) or T.G.C. (1 ml. i.p.). At intervals during tumour growth, four rats from each experimental group and four from the control group were killed and biochemical assays made on the pooled sera from each group (Fig. 1 and 2). Pooling of sera was necessary to obtain sufficient for all the assays. Creatinine levels are not shown as no deviation from control values were observed in any tumour-bearing animals.

The trends of changes in the serum concentrations of the solution estimated associated with growth of either subline were similar but the changes occurred earlier in rats bearing V rather than A subline. For most components (alkaline phosphatase, cholesterol, sodium, potassium, calcium, iron, albumin, globulin, urea uric acid and bilirubin) deviations from normal values did not occur or became apparent only during the terminal stages of tumour growth (Fig. 1). However the serum glucose and SGOT concentration deviated from the normal 8–10 days after inoculation of V subline and 12–15 days for the A subline and became progressively more abnormal until death (Fig. 1 and 2). It seemed possible, therefore, that low glucose levels might be a primary rather than a secondary effect of tumour growth and might be the main cause of death.

In the above experiments, rats had been killed at definite time intervals after inoculation but at indeterminate times before they would have died of tumour. The upward trend in the glucose concentration, during days 16–17 of growth of the V subline, was biased, since the samples contained rats surviving longer than the population mean. To answer the question " were WBP1 (V) and WBP1 (A) killing as a direct result of hypoglycaemia ", the serum glucose concentration at

FIG. 1a.—Changes in serum components following inoculation of WBP1 (A) (○) or WBP1 (V) (△) cells (1 × $10^7$) i.p. The dotted lines show the 95% fiducial limits of the means of values measured from the sera of control rats (T.G.C. solution; 1 ml. i.p.), sampled over the same period. Values are for pooled sera (4 rats).

FIG. 1b.—Changes in serum components following inoculation of WBP1 (A) (○) or WBP1 (V) (△) cells (1 × $10^7$) i.p. The dotted lines show the 95% fiducial limits of the means of values measured from the sera of control rats (T.G.C. solution; 1 ml. i.p.), sampled over the same period. Values are for pooled sera (4 rats).

FIG. 2.—Changes in serum glucose following the inoculation of WBP1 (A) (○) or WBP1 (V) (△) cells (1 × $10^7$) i.p. The dotted lines show the 95% fiducial limits of the means of values measured from the sera of control rats (T.G.C. solution; 1 ml. i.p.), sampled over the same period. Values are for pooled sera (4 rats).

death was required. Rats dying of A or V subline (1 × $10^7$ cells i.p.) were therefore observed until respiration ceased when they were bled. In the hours before death, the fur of the rats was ruffled and they were comatose apart from occasional muscular spasms. Rectal temperatures decreased from 37° C. to 26–32° C. at death (ambient temperature 23–25° C.). In all rats at death, irrespective of the inoculated subline, serum glucose was less than 10 mg. per 100 ml.

*WBP*1 (*X*) *subline.*—It was hoped that studies with this subline, more malignant than WBP1 (V), might accentuate any differences between primary tumour-induced affects and non-specific changes occurring in dying animals. Rats were injected with X cells (1 × $10^7$ i.p.) and pooled sera obtained at intervals for a restricted series of biochemical analyses; in addition, spleens, thymuses, mesenteric lymph nodes and solid abdominal tumours were weighed and erythrocyte PCV's determined (cf. Williams *et al.*, 1968). Although X killed quicker, the amount of tumour present (Table I) and its effect on the PCV (41%) was smaller than that of V (23%) at death (normal PCV: 50%). Serum glucose concentrations at death (11 mg. per 100 ml.) were comparable with the terminal values associated with V and A sublines. Very high SGOT values (> 2000 units per ml.) were observed but changes in other serum components were smaller than those associated with the less malignant sublines (Table II).

TABLE I.—*Organ Weights as a Measure of Visceral Invasion by Sublines WBP1 (X) and WBP1 (V)*

| | $\frac{\text{g. organ}}{\text{g. body weight}} \times 100$ | | |
|---|---|---|---|
| | | Tumour inoculated† rats at death | |
| Organ | Normal rats* | WBP1 (X) | WBP1 (V)* |
| Spleen | 0·18±0·03‡ | 0·60±0·08 | 0·61±0·08 |
| Thymus | 0·08±0·04 | 0·37±0·10 | 0·87±0·36 |
| Solid abdominal tumour | — | 1·69±0·44 | 3·54±0·53 |
| Mesenteric lymph node | 0·19±0·10 | 0·15±0·10 | 0·41±0·20 |

* Data from Williams *et al.*, 1968.

† Rats received WBP1 (X) or WBP1 (V) cells (1 × 10⁷) intraperitoneally.

‡ Fiducial limits (95%).

TABLE II.—*Changes in Serum Components during Growth of WBP1 (X) Subline*

| Serum component (units) | Mean value normal rats ±95% limits | Time (days) post-inoculation* 2 | 4 | 6 | 8 | 10 | 11 | At death |
|---|---|---|---|---|---|---|---|---|
| Glucose (mg./100 ml.) | 217·1±16·9 | 188† | 188 | 210 | 191 | 130 | 23 | 11 |
| SGOT (K units/ml.) | 145·7±20·1 | 245 | 175 | 250 | 450 | 720 | 1200 | 2040 |
| Potassium (mEq./l.) | 6·5±0·5 | 6·3 | 7·1 | 5·7 | 6·3 | 6·8 | 8·2 | — |
| Iron (μg./100 ml.) | 192±24 | 173 | 193 | 185 | 185 | 185 | 173 | — |
| Alkaline phosphatase (units/100 ml.) | 27·0±1·9 | 28 | 26 | 26 | 26 | 22 | 22 | — |
| Urea (mg./100 ml.) | 43·1±2·1 | 42 | 39 | 44 | 37 | 35 | 40 | — |
| Albumin (g./100 ml.) | 3·2±0·12 | 3·2 | 3·0 | 3·0 | 3·0 | 3·0 | 2·8 | — |
| Globulin (g./100 ml.) | 3·6±0·15 | 3·8 | 4·0 | 3·8 | 3·8 | 3·9 | 3·6 | — |

* Rats received WBP1 (X) cells (1 × 10⁷) intraperitoneally.
† Each value was obtained from a pool of sera from 4 rats.

*Serum glucose concentrations in insulin-induced hypoglycaemic death*

To demonstrate that the low glucose concentration in the sera of rats dying from WBP1 tumour could be lethal, hypoglycaemia was induced in normal rats by injection of insulin. The mean serum glucose concentration of seven rats, starved for 48 hours was 180·6 ± 5·5 (s.e.) mg. per 100 ml. Insulin (0·2–0·4 unit) was injected subcutaneously at hourly intervals. Serum glucose concentrations and rectal temperatures were monitored and the general condition of the animals observed. After 4 hours, the mean glucose concentration was 54·6 ± 7·3 mg. per 100 ml. serum. The mean rectal temperature was 33·4 ± 0·4° C. and the rats were lethargic with ruffled fur. Further insulin injections caused a continued decrease in serum glucose concentrations and rectal temperatures. All the rats died between 6 and 11 hours after the first insulin injection with serum glucose concentrations $<$11 mg. per 100 ml. and rectal temperatures of 25–28° C. (ambient 20° C.).

*The effect of injections of glucose on the survival of rats bearing WBP1 (X) subline*

Attempts were made to prolong the life of rats carrying X subline by injecting glucose solutions intraperitoneally. Technical difficulties were encountered which could not be completely overcome. Hypertonic solutions of glucose were toxic. Hence to provide sufficient glucose, large volumes (up to 5 ml. of isotonic glucose solution (5·5%) at 1–4 hour intervals for 5–8 days) had to be injected, with consequent strain on the fluid balance mechanisms of the animals. It proved difficult to maintain the serum glucose concentration in the normal range and in some cases over-administration of glucose produced a fatal hyperglycaemia (> 600 mg./100 ml. serum). Two methods of infusion were tried. (1) Rats were lightly anaesthetized and repeatedly injected intraperitoneally with a syringe; over several days they showed signs of shock and subcutaneous haemorrhage. (2) The peritoneal cavities of rats were cannulated (see methods) 9 days after inoculation of X cells and isotonic glucose solution injected through the cannula either continuously by peristaltic pump or manually at intervals. This avoided the repetitive trauma of method (1) and improved the condition of the rats. Control groups of tumour-bearing rats were infused with saline instead of glucose solution. The serum glucose concentration was monitored as a guide to the injection rates and the serum glucose concentrations at death were determined. Using method (2) a significant ($P < 0{\cdot}001$) prolongation of life was achieved in WBP1 (X)-bearing rats injected with glucose compared with control animals (Table III). The final glucose injection was given at 13·75 days to the 2 surviving rats. Following withdrawal of treatment they rapidly became hypoglycaemic and died 6 and 9 hours later respectively.

TABLE III.—*Prolongation of Survival of WBP*1 *(X)-bearing Rats by Injections of Glucose*

Times (days) of death of individual rats post-inoculation*

| | Controls‡ | | | |
|---|---|---|---|---|
| | Saline infused i.p. by cannula | Cannula inserted; rats restrained; no liquid infused | Unoperated rats | Experimental‡ Glucose infused i.p. by cannula |
| | 10·19 | 10·25 | 10·98 | 11·92 |
| | 10·27 | 11·08 | 11·21 | 13·04 |
| | 10·56 | 11·58 | 11·54 | 14·00 |
| | 11·50 | | | 14·08 |
| Means | 10·63 | 10·97 | 11·24 | |
| | Overall means† = 10·92 ± 0·18 | | | 13·26 ± 0·51 |

* All rats received WBP1 (X) cells (1 × $10^7$) intraperitoneally.
† Means ± standard error; the means of the glucose treated and control rats differ significantly ($P < 0{\cdot}001$).
‡ Serum glucose levels in all rats at death were < 10 mg./100 ml.

*Invasion of organs controlling glucose homeostasis*

In addition to growing as ascites tumours, the WBP1 sublines metastasized widely and might have caused hypoglycaemia by physical destruction of organs controlling glucose metabolism. Histological examination of pancreas and adrenal glands from rats injected intraperitoneally with A, V or X cells (1 × $10^7$) showed progressive infiltration by tumour cells. However, the disruption of normal tissue was no greater after 15–17 days in the rats dying of V subline than in those carrying A subline although the latter group would survive for another 2–3 weeks.

*Terminal serum glucose levels after subcutaneous injection of tumour*

If tumour cells of any of the sublines were injected subcutaneously, a large local solid tumour developed and adjacent lymph nodes were heavily invaded; however, minimal infiltration of the viscera, the pancreas and adrenal glands occurred. Nevertheless, at death, glucose concentrations of $<$ 10 mg. per 100 ml. serum were measured, accompanied by rectal temperatures of 26–32° C.

### DISCUSSION

Studies on the biochemical basis of pathological change are beset by difficulties of distinguishing cause from effect and specific changes from secondary effects of the disease processes. Many changes in serum components observed during growth of the WBP1 tumours, particularly those occurring during the later stages of the disease, were undoubtedly due to the secondary effects. The serum changes observed during growth of the highly malignant WBP1 (X) support this hypothesis. Apart from the decreased glucose and high SGOT values, the changes were less severe than in the more prolonged disease produced by WBP1 (V) and WBP1 (A) and the terminal values observed in all the rats were unlikely to cause death.

Glucose and SGOT changes appeared the most significant. They were detectable early in tumour growth and their onset and severity correlated with the malignancy of the subline. SGOT concentrations are high in diseases producing tissue destruction, particularly of the heart or liver, but as far as we are aware are never fatal. Total tissue damage by invading tumour cells is greatest in rats carrying WBP1 (A) and therefore the higher SGOT values associated with growth of V and X sublines may indicate additional damage by cytotoxic factors possibly produced by the tumour cells. It is of course always possible that the SGOT may be produced by the tumour cells.

Glucose was the only serum component measured that showed the same concentration at death in rats dying from any of the sublines. This concentration (approximately 10 mg. per 100 ml. serum) and the associated severe hypothermia also occurred in rats dying of insulin-induced hypoglycaemia and in patients dying with hypoglycaemic shock (Marble, 1952; Williams, 1962; Himwich, 1951; Kedes and Field, 1964). Although it was not possible to prolong life indefinitely, significant survival was achieved by glucose infusion. The hypoglycaemia caused by the sublines was far more severe than those recorded previously in experimental tumour systems (Victor and Potter, 1938; Silverstein, Wakim, Bahn and Bayrd, 1960).

Thus, hypoglycaemia is the probable cause of death from the WBP1 sublines and the ability of a subline to induce this condition is one important factor determining its degree of malignancy. The early appearance of hypoglycaemia during tumour growth when the amount of tumour was small and metastasis limited suggested that the effect was specifically induced by the tumour and not a secondary change.

Cases of death by hypoglycaemic shock due to extra-pancreatic neoplasms have been reported in man (Lowbeer, 1961; O'Neill and Mikuta, 1970) and several explanations have been suggested (Lowbeer, 1961; Marks and Rose, 1965; Unger, 1966; Silverstein, 1969). Firstly, invasion and destruction of organs controlling glucose metabolism; this seemed an unlikely explanation for the WBP1-induced hypoglycaemia, as the latter appeared to be unrelated to the extent of pancreatic and adrenal invasion and to the distribution of metastases. Secondly, hypoglycaemia may result from over-utilization of glucose by the tumour; this effect may have been significant in the terminal stages of WBP1 growth but is unlikely to be the complete explanation as hypoglycaemia was detected after 8 days for WBP1 (V) and WBP1 (X), when the tumour was only about 1–2% of the total body weight (cf. Williams *et al.*, 1968). Thirdly, hypoglycaemia might result from the disruption of glucose homeostasis by a substance produced by the tumour or by its interaction with normal tissues. Such a substance, for example, might stimulate the action of normal control mechanisms, such as the production of insulin, or function in an insulin-like manner or inhibit the production of glucose by glycogenolysis or gluconeogenesis. The sensitivity to hyperglycaemia induced by glucose administration, of formerly hypoglycaemic rats dying of WBP1 tumour, indicates disturbance of glucose metabolism. The marked correlation between the rates of decrease of serum glucose and increase of SGOT suggests a relationship between the effects. The tumour may damage cells concerned with glucose homeostasis causing hypoglycaemia and the release of intracellular transaminase. Identification of the source of the enzyme may thus lead to an explanation for the hypoglycaemia.

We wish to thank Miss S. M. Christie, Mr. G. G. Nickless, Mr. M. S. Macbeth and Mr. R. Blake for technical assistance and to acknowledge the advice given by Dr. H. B. Stoner and Dr. M. C. Lancaster. The project was supported by a grant from the Birmingham Council of the British Empire Cancer Campaign for Research and by a Science Research Council Studentship award to R.A.K. This work forms part of a Ph.D. thesis submitted to the University of Birmingham by R.A.K.

### REFERENCES

BABSON, A. L., SHAPIRO, P. O., WILLIAMS, P. A. R. AND PHILLIPS, G. E.—(1962) *Clinica chim. Acta*, **7**, 199.
BARTHOLOMEW, R. J. AND DELANEY, A. M.—(1966) *Proc. Aust. Ass. clin. Biol.*, **1**, 214.
BROWN, M. E.—(1961) *Diabetes*, **10**, 60.
FOLIN, O. AND WU, H.—(1919) *J. biol. Chem.*, **38**, 81.
GAMBINO, S. R. AND SCHREIBER, H.—(1964) Technicon Symposium, Paper 54.
HIMWICH, H. E.—(1951) *In* ‘Brain Metabolism and Cerebral Disorders’. Baltimore (Williams and Wilkins) p. 258.
HUANG, T. C., CHEW, C. P., WEFLER, V. AND RAFFERTY, A.—(1961) *Analyt. Chem.*, **33**, 1405.
KEDES, L. H. AND FIELD, J. B.—(1964) *New Engl. J. Med.*, **271**, 785.
KESSLER, G. AND WOLFMAN, M.—(1964) *Clin. Chem.*, **10**, 686.
LOFLAND, H. B. AND CROUSE, L.—(1965) Technicon Symposium, 356.
LOWBEER, L.—(1961) *Am. J. clin. Path.*, **35**, 233.
MARBLE, A.—(1952) *In* ‘A Treatment of Diabetes Mellitus’, edited by Joslin, E. P., Root, H. F., White, P. and Marble, A.—9th Edition. Philadelphia (Lea) p. 307.
MARKS, B. AND ROSE, F. C.—(1965) *In* ‘Hypoglycaemia’. Oxford (Blackwell) p. 187.
MARSH, W. H., FINGERHAUT, B. AND KIRSCH, E.—(1959) *Clin. Chem.*, **5**, 119.
MARSH, W. H., FINGERHAUT, B. AND MILLER, H.—(1965) *Clin. Chem.*, **11**, 624.
NAKAHARA, W.—(1968) *In* ‘Methods in Cancer Research Vol. II’. New York (Academic Press) p. 203.
O'NEILL, R. T. AND MIKUTA, J. J.—(1970) *Obstet. Gynec., N.Y.*, **35**, 287.
SILVERSTEIN, M. N.—(1969) *Cancer, N.Y.*, **23**, 1.
SILVERSTEIN, M. N., WAKIM, K. G., BAHN, R. C. AND BAYRD, E. D.—(1960) *Proc. Soc. exp. Biol. Med.*, **103**, 824.
SMITH, H., WILLIAMS, A. E., LOWERY, R. S. AND KEPPIE, J.—(1968) *Br. J. Cancer*, **22**, 359.

SYLVÉN, B. AND HOLMBERG, B. O.—(1965) *Eur. J. Cancer*, **1**, 199.
UNGER, R. H.—(1966) *Am. J. Med.*, **40**, 325.
VICTOR, J. AND POTTER, J. S.—(1938) *Am. J. Cancer*, **33**, 578.
WEICHSELBAUM, T. E.—(1946) *Am. J. clin. Path.*, **7**, 40.
WHITEHEAD, T. P.—(1968) *Bio-med. Engng*, **3**, 467.
WILLIAMS, A. E., LOWERY, R. S. AND SMITH, H.—(1968) *Br. J. Cancer*, **22**, 367.
WILLIAMS, R. H.—(1962) *In* ' Textbook of Endocrinology ', 3rd edition. Philadelphia (Saunders) p. 715.
YOUNG, D. AND HICKS, J.—(1965) *J. clin. Path.*, **18**, 98.